# An Automated Clinical Laboratory Decision Support System for Test Utilization, Medical Necessity Verification, and Payment Processing

**DOI:** 10.2196/46007

**Published:** 2025-02-11

**Authors:** Safedin Beqaj, Rojeet Shrestha, Tim Hamill

**Affiliations:** 1 Medical Database, Inc Irvine, CA United States; 2 Patient Choice Laboratories Indianapolis, IN United States; 3 Department of Laboratory Medicine University of California San Francisco, CA United States

**Keywords:** clinical decision system, CDSS, laboratory decision system, laboratory testing, test utilization, test ordering, lab test, laboratory, testing, payment, decision-making, user, utilization, processing, decision

## Abstract

Physicians could improve the efficiency of the health care system if a reliable resource were available to aid them in better understanding, selecting, and interpreting the diagnostic laboratory tests. It has been well established and widely recognized that (1) laboratory testing provides 70%-85% of the objective data that physicians use in the diagnosis and treatment of their patients; (2) orders for laboratory tests in the United States have increased, with an estimated volume of 4-5 billion tests per year; (3) there is a lack of user-friendly tools to guide physicians in their test selection and ordering; and (4) laboratory test overutilization and underutilization continue to represent a pervasive source of inefficiency in the health care system. These inappropriate test orders not only lead to slower or incorrect diagnoses for patients but also add a significant financial burden. In addition, many ordered tests are not reimbursed by Medicare because they are inappropriate for the medical condition or were ordered with the incorrect International Statistical Classification of Diseases and Related Health Problems, Tenth Revision diagnostic code, not meeting the medical necessity. Therefore, current clinical laboratory test ordering procedures experience a quality gap. Often, providers do not have access to an appropriate tool that uses evidence-based guidelines or algorithms to ensure that tests are not duplicated, overused, or underused. This viewpoint lays out the potential use of an automated laboratory clinical decision support system that helps providers order the right test for the right disease and documents the right reason or medical necessity to pay for the testing.

## Introduction

Laboratory testing plays a key role in clinical decision-making and physician orders for laboratory tests are increasing [1,2]. It is estimated that at least 20% of the 4-5 billion lab orders submitted annually in the United States are inappropriate. Studies have shown that overutilization and underutilization of laboratory tests occur 20.6% and 44.8% of the time, respectively [3]. This inappropriate testing not only leads to incorrect or delayed diagnoses but also significantly adds a financial burden on the health care system. This situation is expected to worsen as the available lab tests menu grows, especially in the areas of molecular diagnostics and genetic testing. Due to a lack of physician test information, education, and insurance coverage questions, ordering less effective and sometimes obsolete tests over newer tests that are more sensitive and specific remains a major problem [4]. This inappropriate testing not only led to incorrect or delayed diagnoses but also significantly added financial burden. The situation is expected to get worse as the number of lab tests is growing, especially in molecular diagnostics and genetic testing. The introduction of an automated clinical decision support system (CDSS) that guides physicians to order the most appropriate test(s) for their patients while simultaneously providing both medical necessity requirements and applicable diagnostic codes will be a vital tool to improve test ordering and reimbursement efficiency.

Medicare and commercial health-care plans all require that ordered tests are accompanied by appropriate *International Statistical Classification of Diseases and Related Health Problems, Tenth Revision* (*ICD-10*) diagnostic codes that meet medical necessity rules. These requirements make it a complex process for providers to decide which tests to order, provide diagnostic information, and obtain previous authorization if required, so that test bills and payments are efficient and timely [5,6]. Given the rapidly growing demand for tests, especially molecular and genetic testing, the lack of a reliable laboratory CDSS will compound this already complex process.

When physicians fail to select and order the most appropriate test(s) based on the patient’s health condition and further fail to provide the proper diagnostic codes to support medical necessity, laboratory billing will most certainly fail. The patient may then be held responsible for the laboratory charges and the laboratory will be caught in the middle of disagreements between the insurance company, the treating physician, and the patient in determining the responsible party for the laboratory charges. Ideally, every test ordered and procedure performed by the lab should be paid or reimbursed by health insurance. However, many ordered tests are not reimbursed, primarily due to a lack of medical necessity. This issue arises from ordering the wrong test that does not meet medical necessity criteria or failing to provide the correct diagnostic code for the disease or health condition. Therefore, the current clinical laboratory test-ordering procedures suffer from a quality gap and require an automated system to address this issue.

This viewpoint discusses the use of an automated laboratory CDSS that helps providers order the right test for the right disease and documents the right reason or medical necessity to pay for the testing.

## Inappropriate Test Ordering

Inappropriate testing encompasses both overutilization and underutilization, both of which can affect quality patient care and health care expenditures. Overutilization includes tests that are ordered but not indicated, tests that are ordered at the incorrect time in the clinical course, or tests that are ordered too frequently. Conversely, underutilization refers to tests that are indicated but not ordered, or those that are not ordered at the appropriate time to positively impact patient care [2,5]. Both can have an adverse impact on the quality of patient care and health care costs because of downstream consequences such as additional diagnostic testing, repeat testing, imaging, prescriptions, surgeries, or prolonged hospital stays. It is estimated that more than three hundred million patients visit the laboratory annually and that at least 23 million of these patients are affected by inappropriate test ordering and test interpretation. The reports on the Commonwealth Fund Survey of Public Views of the US Health Care System (2012) found that more than 23% of laboratory tests ordered by physicians were duplicated or repeated, which increases the cost of care by further delaying or confusing the patient’s diagnosis and care. It is also reported that overutilization and underutilization of laboratory tests occurred 20.6% and 44.8% of the time, respectively [2].

In 2011, a survey conducted by the Centers for Disease Control and Prevention among primary care physicians found that 14.7% had uncertainty in selecting and ordering the correct test and 8.3% had difficulty interpreting tests [7]. Physicians’ lack of access to information about the availability of specific tests, the limitations of tests, and insurance coverage might play a major role in the underutilization of tests, whereas medical malpractice, selecting obsolete testing, and use of certain aspects of computerized provider order entry are documented factors leading to overutilization [3,4].

## Economic Impact of Test Misutilization

Expenditures for health care in the US were approximately $4.1 trillion in 2020, which is an increase of 9.7% from 2019, and it accounts for 19.7% of the total gross domestic product. If the trends continue, health care costs are projected to increase to $6.2 trillion by 2028 [7]. Hospital and clinical service expenditure also showed rapid growth in 2020 and accounted for approximately $2.08 trillion of the total cost of health care. Although laboratory testing accounts for only a fraction of health care expenditures, 94% of objective and structured data in the electronic medical record (EMR) are obtained from a clinical laboratory [8]. Moreover, it is estimated that 60%-70% of all clinical decisions are based on the results of laboratory testing [9,10]. Considering 60% as the rate of influence on the clinical decision, it can be estimated that $1.2 trillion of health care spending is influenced by laboratory testing. Therefore, inappropriate testing not only leads to incorrect or delayed diagnoses but also significantly adds financial burden. 4-5 billion tests are performed annually in the United States. Unfortunately, it is estimated that at least 20% of the lab orders submitted are inappropriate [2]. The situation is expected to get worse as the number of esoteric lab tests is growing, especially in the areas of molecular diagnostics and genetic testing.

Ideally, every test performed by the laboratory should be reimbursed. However, many billed tests are not reimbursed due to a lack of documentation ensuring medical necessity. In many cases, the denial of the reimbursement is due to the submission of improper diagnostic code(s) for the disease or health problem being tested for. Therefore, current clinical laboratory test ordering procedures experience an information gap and there is an urgent need for an automated system to improve test utilization for economic sustainability in health care.

## Need for Clinical Decision Support System in Clinical Laboratory

Selecting and obtaining authorization for appropriate medical tests is an ongoing and growing challenge in many specialties, including radiology, cardiology, pulmonology, and pharmacology. With typical radiology and diagnostic imaging costs higher than those for laboratory testing, the US government has prioritized approval of a reimbursement reward system for insurance providers that use a CDSS to improve imaging utilization. For example, there is a 2015 “Advanced Imaging Bill” which mandates that government-approved imaging services will only be reimbursed if the insurance claim confirms that appropriate-use criteria were consulted or a CDSS was used. The bill also recommends the use of CDSS for other diagnostic test ordering, if available [5,11,12].

CDSS is currently also available in cardiology, medication management, oncology, and urology. These broad and growing applications along with expansive and expensive specialized lab testing strongly indicate that there is a substantial need for an expert laboratory CDSS to aid health care providers, care managers, and payers in selecting, ordering, and approving laboratory tests and reducing inappropriate testing.

Currently, there are some partially developed and semimanual lab CDSSs that help physicians order laboratory tests; however, these approaches are provider-driven and require inconvenient interactive user questions to access the information needed [10]. Unlike radiology CDSSs, these systems do not provide any scoring system for tests based on medical evidence, clinical relevancy, or medical necessity. Incorporating a scoring system based on test indications and providing information on supportive diagnostic codes can help automate the laboratory test ordering process and has positive impacts on test utilization, medical necessity documentation, claim verification, and payment processing. These developments strongly indicate that there is also a substantial need for a laboratory CDSS to help health care providers in selecting and ordering the appropriate laboratory tests, reduce inappropriate testing, aid providers in easier and more automated payment processing, and finally get better and on-time health care to patients [4,5,13,14].

## Solution to This Problem

A potential solution is to develop a laboratory CDSS that will aid providers in selecting and ordering the right diagnostic tests with which to manage patient health care. The CDSS will help laboratories process the order, process the sample, and report accurate results on-time delivery to the ordering provider. The CDSS will provide information regarding the appropriate diagnostic *ICD-10* code(s) to meet the medical necessity. The CDSS will also provide a medical evidence-based scoring system based on clinical utility. A CDSS that provides the testing indication(s) to complement the provider’s notes and is electronically interfaced with EMRs, electronic hospital records, and billing systems to automate processes like medication management and radiology CDSS is desirable [4,5,14].

Laboratory Decision System (LDS), developed by Medical Database Inc, is one of the available automated CDSS. LDS is an algorithm-based test selection and ordering database for physicians, health care providers, insurance and managed care companies, and billing services. LDS is expertly developed to help system users understand, select, order, and use laboratory tests for disease diagnosis and management using evidence-based guidelines and industry best practices. The system uses our proprietary scoring system developed by our editorial board (60 pathologists and PhDs), designed to rank testing recommendations based on disease, clinical relevance, medical necessity, and testing indication. Each time an order is placed via the LDS platform, it automatically includes the appropriate diagnostic *ICD-10* code and Local Coverage Determinations (LCD) or National Coverage Determinations (NCD) to meet medical necessity for reimbursement. Included in the robust database are all commercially available tests (over 2300 diagnostic tests), including genetic and proprietary tests [5].

The LDS rates and scores potential tests for any given disease and assigns an easily interpretable numerical and color-coded score based on clinical relevance, medical necessity, and testing indication. Tests with scores of 5 or above (10 being the highest score) meet medical necessity, while those with scores of 4 or less do not. LDS also follows Medicare’s medical necessity guidelines by using testing indications such as “initial testing indication” to allow providers to better characterize the patient’s disorder based on initial test results before ordering overly complex and expensive tests. Appropriate tests use indication labels, for example, diagnostic, disease management, monitoring, and alternative tests, categorizing each test with the right indication or reason for testing to avoid using providers’ charts and notes that make it difficult to automate the system [4,5]. When assessing the effectiveness of LDS in improving test utilization and reimbursement with 96,170 laboratory requests comprising 374,423 test orders from a reference laboratory, 44,671 tests were accompanied by *ICD-10* that are described by Medicare as “never covered” because of the lack of a system to check or support the medical necessity of each order. A total of 160,449 tests were subject to a Medicare policy review from which 112,400 tests met coverage criteria, and 48,049 tests did not. These orders were then reevaluated using LDS. Of the original test order sample, 91.5% had an associated LDS score. Of these scored tests, 47.8% met coverage and 43.73% failed to meet coverage, according to the LDS Ranking System. Importantly, LDS provided recommendations for alternative diagnostic *ICD-10* codes or tests which could have aided physicians in choosing a more appropriate test or submitting a different *ICD-10* diagnostic code to meet medical necessity. Around 96.4% have an alternative *ICD-10* code or a test score above 5, meeting medical necessity. The LDS system recommended 80.5% which would meet Medicare policies, demonstrating that the LDS system would correct inappropriate orders if used as a testing utilization management system [5]. However, more systemic testing of the platform is needed to evaluate the effectiveness of test utilization, medical necessity verification, and payment processing.

**Figure 1:**
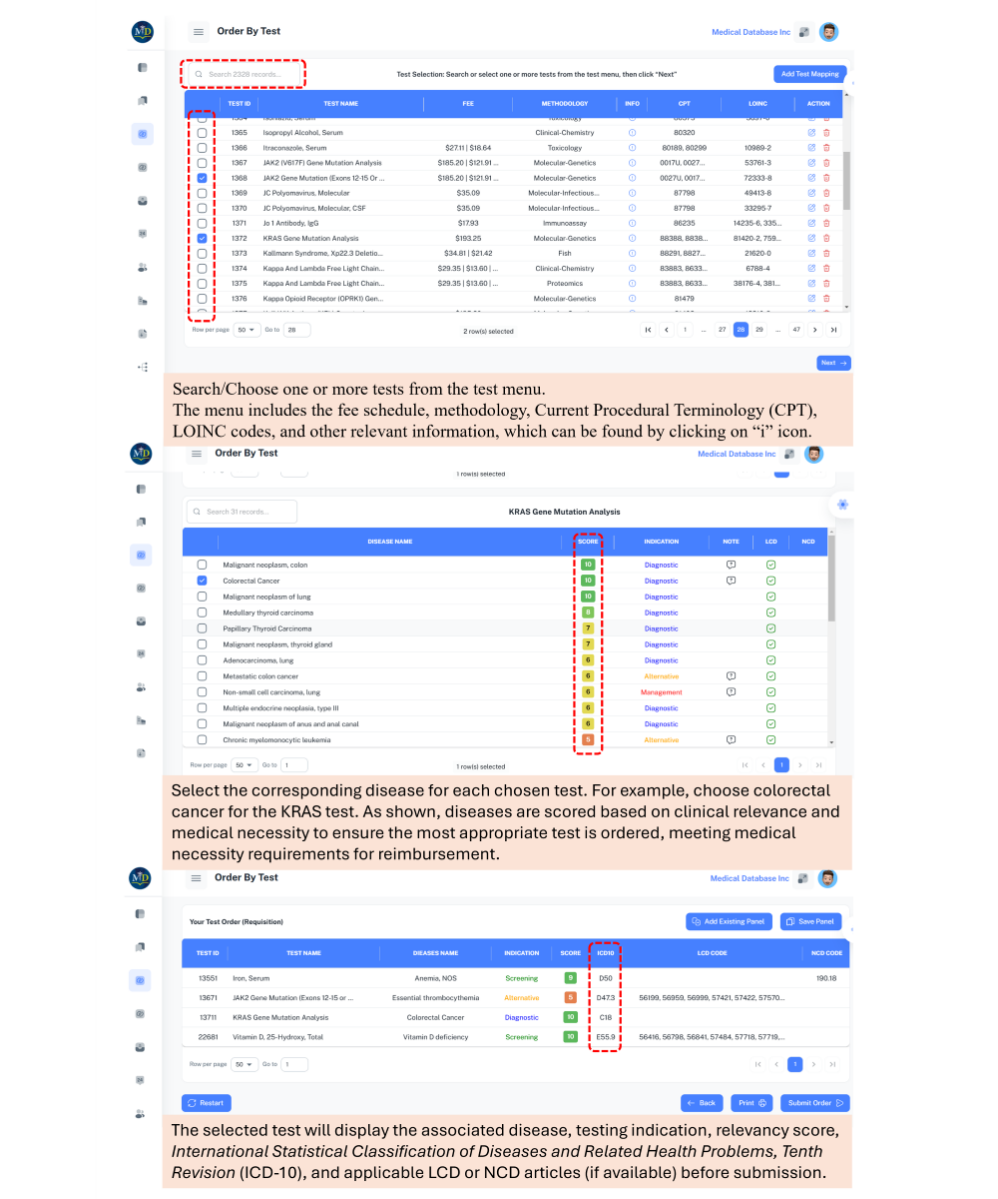
Test ordering using LDS automates the selection of appropriate tests based on clinical relevance and integrates the International Statistical Classification of Diseases and Related Health Problems, Tenth Revision and LCD codes to facilitate reimbursement. LCD: Local Coverage Determinations; LOINC: Logical Observation Identifiers Names and Codes; NCD: National Coverage Determinations.

Since the LDS platform has been built to interface with EMRs, electronic hospital records, and laboratory information systems (LIS), the content can be accessed directly through these systems, allowing orders to be sent directly to laboratories for testing coordination. Accordingly, when using the LDS platform, every test ordered will automatically include a medical necessity score, the correct testing indication, and the appropriate *ICD-10* and CPT codes, all of which also support adjudication for bill payment. An outline of the use of computerized provider order entry of LDS is elucidated in Figure 1. In addition, each test ordered through the LDS platform will provide testing indications to support the purpose of the testing, thereby reducing manual submission of “medical necessity” review data, including reasons for test ordering (scripts, notes, charts, etc) and adding system automation with lower costs, faster throughput, and higher performance [4,15].

## Conclusion

In conclusion, there is a clear and immediate need for an LDS system similar to that which is used in radiology and medication management, which can aid providers in selecting the right test for each disease or condition while assigning the correct *ICD-10* code, right Local Coverage Determinations and National Coverage Determinations to meet the medical necessity and right testing indication covering the reason and use of ordered test(s). The available LDS system developed by Medical Database, described in this viewpoint study may assist providers in making appropriate utilization decisions while also supporting laboratories in reimbursement and streamlining claim verification for payers, all of which combined will potentially make the laboratory industry and overall health care more efficient and cost-effective.

## Abbreviations

CDSS: clinical decision support system
EMR: electronic medical record
ICD-10: International Statistical Classification of Diseases and Related Health Problems, Tenth Revision
LDS: Laboratory Decision System
